# HIV prevalence and incidence among men who have sex with men and transgender women in Bangkok, 2014–2018: Outcomes of a consensus development initiative

**DOI:** 10.1371/journal.pone.0262694

**Published:** 2022-01-21

**Authors:** Frits van Griensven, Nittaya Phanuphak, Chomnad Manopaiboon, Eileen F. Dunne, Donn J. Colby, Pannee Chaiphosri, Reshmie Ramautarsing, Philip A. Mock, Thomas E. Guadamuz, Ram Rangsin, Kanya Benjamaneepairoj, Panus Na Nakorn, Ravipa Vannakit, Jan Willem de Lind van Wijngaarden, Matthew Avery, Stephen Mills

**Affiliations:** 1 Institute of HIV Research and Innovation and Center of Excellence in Transgender Health, Chulalongkorn University, Bangkok, Thailand; 2 Department of Epidemiology and Biostatistics, University of California, San Francisco, CA, United States of America; 3 Thailand Ministry of Public Health–U.S. Centers for Disease Control and Prevention Collaboration, Nonthaburi, Thailand; 4 Division of Global HIV and Tuberculosis, U.S. Centers for Disease Control and Prevention, Atlanta, GA, United States of America; 5 Division of HIV/AIDS Prevention, U.S. Centers for Disease Control and Prevention, Atlanta, GA, United States of America; 6 Division of AIDS, TB and STI, Bangkok Metropolitan Administration, Bangkok, Thailand; 7 Faculty of Social Sciences and Humanities, Mahidol University, Salaya, Thailand; 8 Phramongkutklao College of Medicine, Bangkok, Thailand; 9 FHI 360, Bangkok, Thailand; 10 United States Agency for International Development, Regional Development Mission for Asia, Bangkok, Thailand; 11 Independent Researcher, Bangkok, Thailand; International AIDS Vaccine Initiative, UNITED STATES

## Abstract

To reach its goal of ending AIDS by 2030, Thailand has adopted antiretroviral treatment as prevention and HIV pre-exposure prophylaxis for men who have sex with men (MSM) and transgender women (TGW) as its core HIV control strategy. However, in the absence of reliable epidemiologic indicators, the impact of these policies on the course of the HIV epidemic in these groups remains unknown. To help answer this question, we formulated an HIV epidemic consensus initiative for Bangkok, Thailand, to analyze epidemiologic and program data and reach agreement between experts and stakeholders on the evolving state of the HIV epidemic among MSM and TGW. A customized Delphi process was used to consult and consolidate viewpoints of experts and stakeholders. Experts presented and discussed HIV prevalence and incidence data from recent and ongoing studies among MSM and TGW in Bangkok (2014 to 2018) during a meeting with stakeholders representing government, donors, and civil society. Agreement about the course of the HIV epidemic among MSM and TGW was attained by voting consensus. Based on presented data, meeting participants agreed that HIV prevalence and incidence had decreased among Bangkok MSM from 2014 to 2018. Despite these declines, HIV prevalence and incidence were found to remain high. This was particularly the case among younger MSM. Participants agreed that there was no evidence for a decrease in HIV prevalence and incidence among Bangkok TGW. Introduction of antiretroviral treatment as prevention and HIV pre-exposure prophylaxis may have contributed to these declines. However, HIV prevalence and incidence remained high, and no signs of a decrease were reported among Bangkok TGW. At the current rate of new HIV infections in MSM and TGW, Thailand will not reach its goal of ending AIDS by 2030. This HIV consensus initiative may serve as a model for building agreement and advocacy on epidemiologic and program data and their implications for a large metropolitan city.

## Introduction

Thailand has made dramatic and clear achievements in controlling its HIV epidemic, and is internationally recognized for high-quality HIV research and programming [1]. In 1991, Thailand launched the “100% condom use in commercial sex program”, widely believed to be the first and possibly the only national program achieving successful control of a generalized HIV epidemic [2]. In 1996, it was the first country to start hosting its own clinical trials of more affordable antiretroviral (ARV) regimens for the prevention of mother-to-child HIV transmission [3]. By 2001, close to 100% of pregnant Thai women were HIV tested and offered ARVs for them, their newborns, and partners; and in 2016 Thailand became the first country in Asia to have eliminated new HIV infections among children [4].

Thailand again served as a role model by introducing its National Access Program to ARV drugs for people with HIV in 2001, the first non-high-income country doing so [5]. In 2006 and 2007 Thailand issued compulsory licenses for the import and local production of ARV’s to ensure continued access to these life-saving drugs for Thais with HIV infection, despite the looming threat of punitive economic measures [6].

Notwithstanding these successes, integrated behavioral and biological surveillance (IBBS) among men who have sex with men (MSM) in Bangkok in 2003 found an HIV prevalence of 17.3%, further increasing to over 30% by 2005 and 2007 [7]. In biennial follow-up conducted through 2014, HIV prevalence among MSM continued to fluctuate around 25% to 30%, indicating ongoing HIV transmission at the population level [8]. This high and increasing HIV prevalence was later confirmed from several other sources, notably the Thai Red Cross Anonymous Clinic (TRCAC), where 29.1% of 3485 MSM attending HIV testing and counseling (HTC) services from 2006 to 2009 were HIV-infected [9], and at the Silom Community Clinic (SCC), with 28.3% of 4762 MSM attending similar services between 2005 and 2011 testing HIV seropositive [10]. High HIV incidence rates among MSM were also reported: among 8176 men seeking HIV re-testing at the SCC during 2006–2013, HIV incidence density was 5.5 per 100 person-years (PY) [10], and among 1744 MSM enrolled in a cohort study at the same clinic, it was 5.3 per 100 PY [11].

Transgender women (TGW) were first included in IBBS in 2005 [12], and inconsistently and in varying numbers and locations in subsequent years. The HIV prevalence among TGW in Bangkok was 11.5% in 2005, 10.0% in 2009 and 9.9% in 2014 [8]. HIV prevalence in underlying age groups varied, with more than 20% of TGW of 30 years and older testing HIV positive in 2014 [1, 11]. No other systematic HIV prevalence and incidence data were available for TGW in Bangkok.

Receptive anal intercourse, lack of condom use, high levels of sexual partner turnover, drug use during sex, and finding casual partners via the internet have all been found to be associated with higher HIV prevalence and incidence among Bangkok MSM and TGW [13–15]. In addition, fewer than half of MSM and TGW reported to have ever been HIV tested, and even fewer said to have actual knowledge of their current HIV status [8, 16].

The HIV epidemic among MSM and TGW continued to unfold during the beginning of the past decade. During this period, however, two highly effective biomedical interventions emerged from phase 3 efficacy trials: 1) ARV HIV pre-exposure prophylaxis, or PrEP [17], and 2) antiretroviral treatment (ART) as prevention (TasP) [18]. The first pertains to a daily (or intermittent) oral dose of two ARVs (tenofovir disoproxil fumarate (TDF) and emtricitabine (FTC)) as chemoprophylaxis against infection, and the second refers to the reduced risk of onward transmission after the initiation of ART with subsequent reduction in HIV viral load. Concurrently, several promising laboratory techniques became available, such as self, oral, rapid, and combined antigen and antibody HIV testing [19–21].

As part of its commitment to ending AIDS by 2030 [22], the Thai Government included TasP and PrEP for key populations as priorities in its National AIDS Strategic and Operational Plans since 2014–16 [23, 24]. Within the framework of these plans, several government, non-government and community-based organizations and their national and international partners and funders initiated a variety of programmatic activities to increase community involvement, promote HIV testing, and expand treatment and care services among MSM and TGW. Some activities, such as increased HIV testing and early treatment, began in 2012 and were scaled-up over the ensuing years. In 2014, national HIV treatment guidelines were expanded to recommend ART for all HIV-infected Thais, regardless of CD4+ cell count [25]. Later that year, the first fee-based HIV PrEP program was initiated, followed by several other stand-alone and integrated PrEP demonstration projects [26]. By 2018, the Thai Food and Drug Administration approved PrEP for HIV prevention and financing by the National Health Security Office was authorized in 2019 [27]. However, gradual nation-wide scale up as part of the country’s Universal Health Coverage Scheme only started in 2020 [28].

While it may be assumed that these activities increased access to and uptake of HIV testing, earlier initiation of treatment, and use of PrEP, their overall impact on the course of the HIV epidemic among MSM and TGW in Bangkok remains unknown. There is no existing structure to aggregate research, surveillance, and program data into reliable HIV epidemiologic indicators. For this reason, we conceptualized the Bangkok HIV epidemic consensus initiative to: (1) establish a panel of local experts in HIV strategic information; (2) select, review, and discuss recent published and unpublished local epidemiological data (HIV prevalence and incidence) for presentation and deliberation with stakeholders; and (3) reach consensus between experts and stakeholders about the current and possible future course of the HIV epidemic among MSM and TGW in Bangkok. The outcomes of this process are expected to identify new or ongoing HIV prevention challenges and guide appropriate interventions among MSM and TGW in the Thai capital.

## Methods

### Delphi technique

This consensus-building exercise applied a modified Delphi process to reach consensus between a panel of experts from government agencies and academia with stakeholders from non-government and community-based organizations about the current and possible future course of the HIV epidemic among MSM and TGW in Bangkok. Originally, the Delphi technique was developed as an iterative process of soliciting expert opinions for the purpose of reaching consensus about a complex problem and has been used by metropolitan areas to reach agreement on multiple and complex HIV epidemiologic data [29, 30]. It is based on the paradigm that agreement is possible, and that consensus is a valuable outcome of this process. For example, the San Francisco Department of Public Health used a modified Delphi method to bring together researchers, service providers, public health officials, epidemiologists, and community representatives to review existing data and to agree on the best estimates of HIV prevalence and incidence for San Francisco, an early HIV epicenter. These data were called the "HIV Consensus Estimates" and were produced multiple years to inform a diverse set of key HIV epidemiologic and surveillance data and to advocate for funding support at the local, state, and national level for interventions [31, 32].

### Selection of experts and stakeholders

Local representatives from government, non-government, and private organizations who have served in in HIV epidemiology, strategic information, and prevention among MSM and TGW (e.g., researchers, scientists, monitoring and evaluation specialists, interventionists) were approached to take part in the expert panel. Stakeholders were invited from non-government and community-based organizations and civil society groups involved in HIV prevention, treatment, and equal rights advocacy for MSM and TGW.

### Consensus meeting

Published and unpublished recent HIV prevalence and incidence data (2014 to 2018) among MSM and TGW were presented by experts during a consensus meeting with stakeholders in April 2019. Where possible, experts were asked to disaggregate data by calendar year and age. Following presentation and deliberation of data, six items regarding the epidemiology of HIV infection among MSM and TGW were presented for voting consensus (agree/disagree). Items were informed and prepared by the expert panel after reviewing available data. A copy of the set of voting items is included as S1 Appendix with this paper. Votes were casted electronically using an online survey instrument (Survey Monkey, San Mateo, CA, USA). The project team, interpreters and observers were not eligible to vote. For each item, consensus was considered achieved if two-thirds of the votes agreed with the content of the item.

### Ethical review

Study protocols of published and unpublished research data presented at the consensus meeting were approved by the Institutional Review Boards of Chulalongkorn University, Bangkok, Thailand (Key Population-led Community Test and Treat Study [33], and Acute HIV Infection Study [34]), Walter Reed Army Institute of Research, Bethesda, United States (Acute HIV Infection Study [34]), Thai Ministry of Public Health and US Centers for Disease Control and Prevention (Test, Treat and Prevent Program [35] and the Prevention Study [36]), and Mahidol University, Salaya, Thailand (Bangkok Online HIV Testing in Young MSM Study [37]). Routine data collected as part of IBBS, and HTC services were not considered research and therefore exempt from ethical review [7, 8, 10].

## Results

### Men who have sex with men

#### HIV prevalence

**Fig 1:**
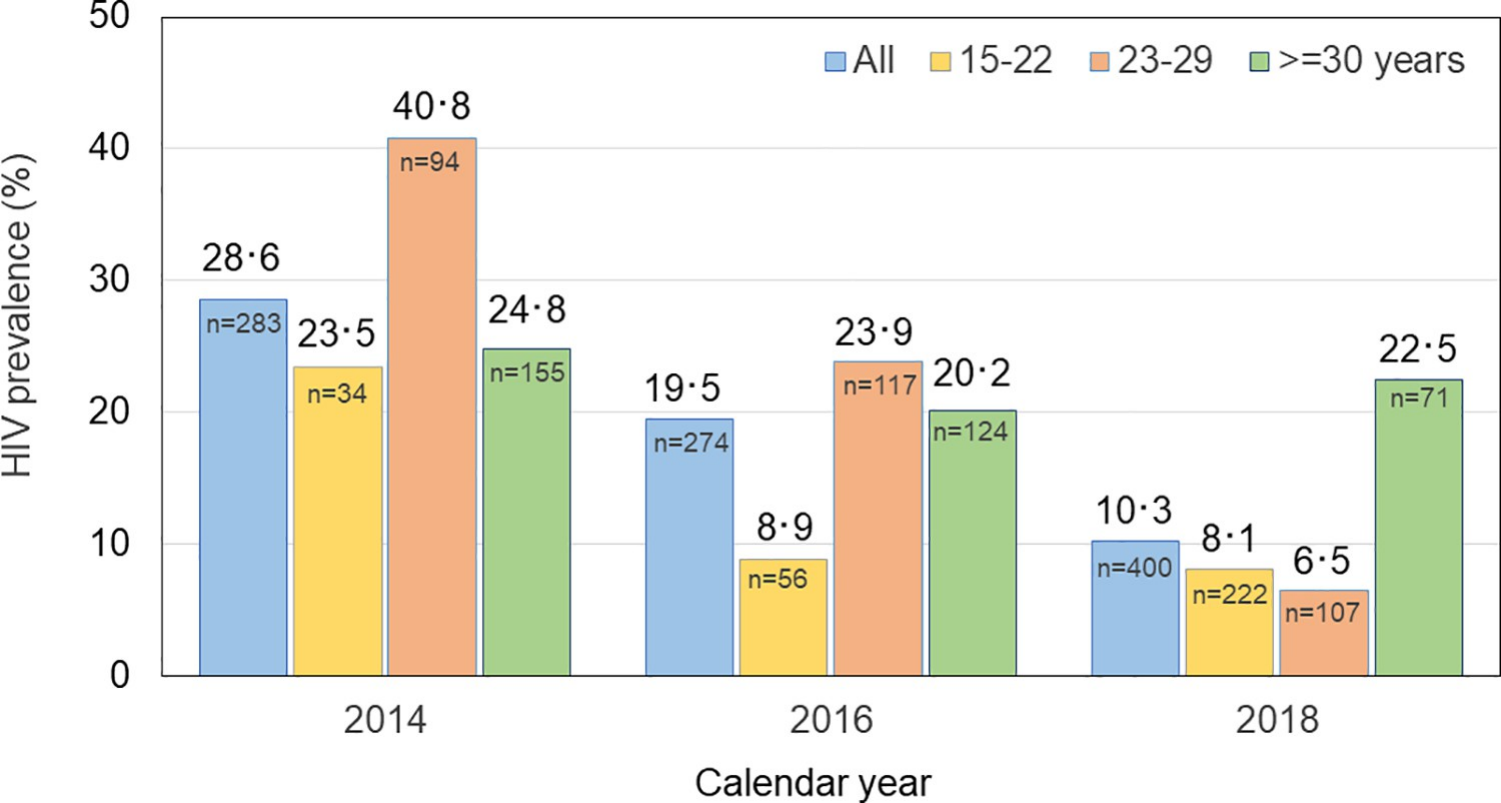
HIV prevalence among men who have sex with men, integrated behavioral and biological surveillance, Bangkok, 2014–2018, by age group.

*HIV prevalence in cross-sectional studies*. Extensive HIV prevalence data were available for MSM in Bangkok. Information was presented from several cross-sectional studies, with HIV prevalence ranging from 7.2% (100/1394) in the Bangkok Online HIV Testing in Young MSM Study [37], to 21.4% (176/824) in the Bangkok arm of the Key Population-led Community Test and Treat Study [33]. High HIV prevalence (20.5%) was also reported from the two Bangkok sites of the Test, Treat and Prevent Program [35]; HIV prevalence being highest among 22- to 29-year-olds (59/208 or 28.4%), followed by those 30 years and older (25/151 or 16.6%), and those 21 years and younger (24/168 or 14.3%) (C Manopaiboon, personal communication). Similarly, data were presented from IBBS, conducted biennially by the Division of AIDS, TB and STI of the Bangkok Metropolitan Administration [8]. Using venue-day-time sampling, men were enrolled at different days and times from physical locations (such as bars, discos, saunas and public parks) in Bangkok where MSM congregate for socializing or seeking sexual partners [38]. In 2014, HIV prevalence was 23.5% among 15- to 22-year-olds, 40.8% among 23- to 29-year-olds and 24.8% among those 30 years and older. Four years later, the prevalence among 15- to 22-year-olds had decreased to 8.1%, among 23- to 29-year-olds to 6.5% and had remained stable among those of 30 years and older, ~23%. Consequently, overall HIV prevalence among MSM included in IBBS declined from 28.6% in 2014 to 10.3% in 2018 (Fig 1). It was reported, however, that the proportion of young MSM (≤22 years) included in IBBS increased from 12.6% (n = 56) in 2014 to 55.6% (n = 222) in 2018 [8].

*HIV prevalence at HIV testing sites*. Cross-sectional HIV prevalence data among MSM attending HTC services in Bangkok were summarized during the meeting. Among 1465 MSM attending HTC at the site of the Prevention Study, the 2016 cumulative HIV prevalence was 21.6% (317/1465) [36]. In age-disaggregated analysis it was 23.9% among those ≤21 years (67/269), 24.5% among those 22–29 years (148/603) and 17.2% among those ≥30 years (102/593) (C Manopaiboon, personal communication).

**Fig 2:**
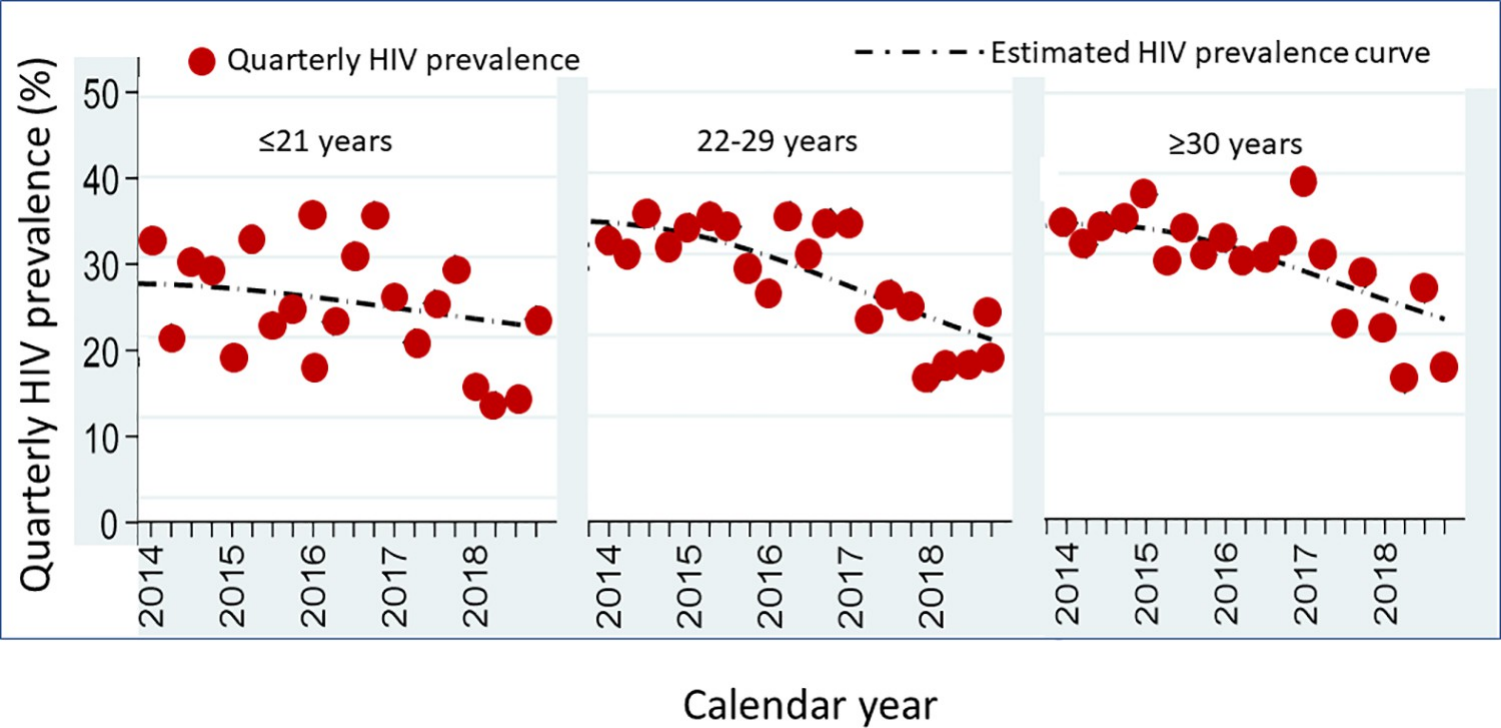
Quarterly HIV prevalence among men who have sex with men attending HIV testing and counseling services at the Silom Community Clinic, Bangkok, 2014–2018, by age group.

Data were also presented from TRCAC, the largest HTC facility in Bangkok and visited by several thousands of clients yearly. In 2014, 6348 new MSM clients attended the clinic, increasing to 7009 by 2018. HIV prevalence among these MSM decreased from 25.6% in 2014 to 17.1% in 2018 (Table 1) (N Phanuphak, personal communication). The SCC, a different HTC setting, has been serving more than 14000 MSM since 2005. In 2014, 1626 new MSM clients were seen compared to 724 in 2018; HIV prevalence in these men decreased from 31.4% in 2014 to 17.1% in 2018 (Table 1) [39]. Quarterly HIV prevalence disaggregated by age group from 2014 to 2018 was reported (Fig 2). HIV prevalence significantly decreased in those aged 22 to 29 years and aged 30 years and older; HIV prevalence among in those aged < = 21 years did not significantly change [39].

**Table 1:** HIV prevalence among men who have sex with men attending HIV testing and counseling services at the Thai Red Cross Anonymous Clinic and Silom Community Clinic Bangkok, 2014–2018.

Venue	Thai Red Cross Anonymous Clinic			Silom Community Clinic		
Calendar year	Number HIV tested	Number HIV reactive	HIV prevalence	Number HIV tested	Number HIV reactive	HIV prevalence
2014	6348	1625	25.6	1626	511	31.4
2015	6690	1481	22.1	1650	512	31.0
2016	7373	1374	18.6	1695	519	30.6
2017	7387	1408	19.1	1285	342	26.6
2018	7009	1249	17.8	724	124	17.1

#### HIV incidence

*HIV incidence estimates from cross-sectional studies*. Directly observed estimates of HIV incidence among MSM are difficult to obtain, especially among young MSM [40]. In these men, HIV incidence is recent and (if the start of sexual risk behavior is known) can be derived from HIV prevalence as follows: (number of prevalent infections / (sum of (current age ─ age at start of anal intercourse)). This approach was recently scientifically validated [40], and applied to data from young MSM enrolled in IBBS in Bangkok from 2014 to 2018 [8]. Estimated HIV incidence density in this population was calculated 7.6 (8/105) per 100 person years (PY) in 2014, 2.4 (5/214) in 2016 and 2.6 (17/665) in 2018 (F van Griensven, personal communication).

The presence of acute HIV infection (during the first month after acquisition) can be derived from sequential emergence of serological markers while the person is still testing HIV seronegative by conventional laboratory methods [34]. Therefore, laboratory based acute HIV infection diagnosis can be used as proxy for incident HIV infection. In the Acute HIV Infection Study, TRCAC has been testing all new and returning HIV seronegative MSM clients for the presence of acute HIV infection for more than a decade [9, 41]. In 2014, 9013 HIV seronegative tests were performed with an acute HIV infection rate of 12.2 per 1000. This number of tests increased from 9013 in 2015 to 13511 by 2018 (Table 2). From these data it can be derived that HIV incidence may have fallen by more than 50% among MSM attending HTC at TRCAC (D Colby, personal communication).

**Table 2:** Acute HIV infection rate among men who have sex with men attending HIV testing and counseling services at the Thai Red Cross Anonymous Clinic, Bangkok, 2014–2018.

Calendar year	Number of HIV tests	Number with acute HIV infection	Acute HIV infection rate (x1000)
2014	9013	88	12.17
2015	8436	95	13.61
2016	13272	93	6.38
2017	13652	86	7.19
2018	13511	55	4.59

*HIV incidence in follow-up studies*. Currently, few observational follow-up studies assess HIV incidence among Bangkok MSM. Data were presented from the Bangkok sites of the Test, Treat, and Prevent HIV Program [35], (7/179.9 or 3.9 per 100 PY) [42], and the Prevention Study [36], (30/421.5 or 7.1 per 100 PY) (C Manopaiboon, personal communication) and Bangkok Online HIV Testing in Young MSM Study (3/29.5 or 10.2 per 100 PY) [37].

**Fig 3:**
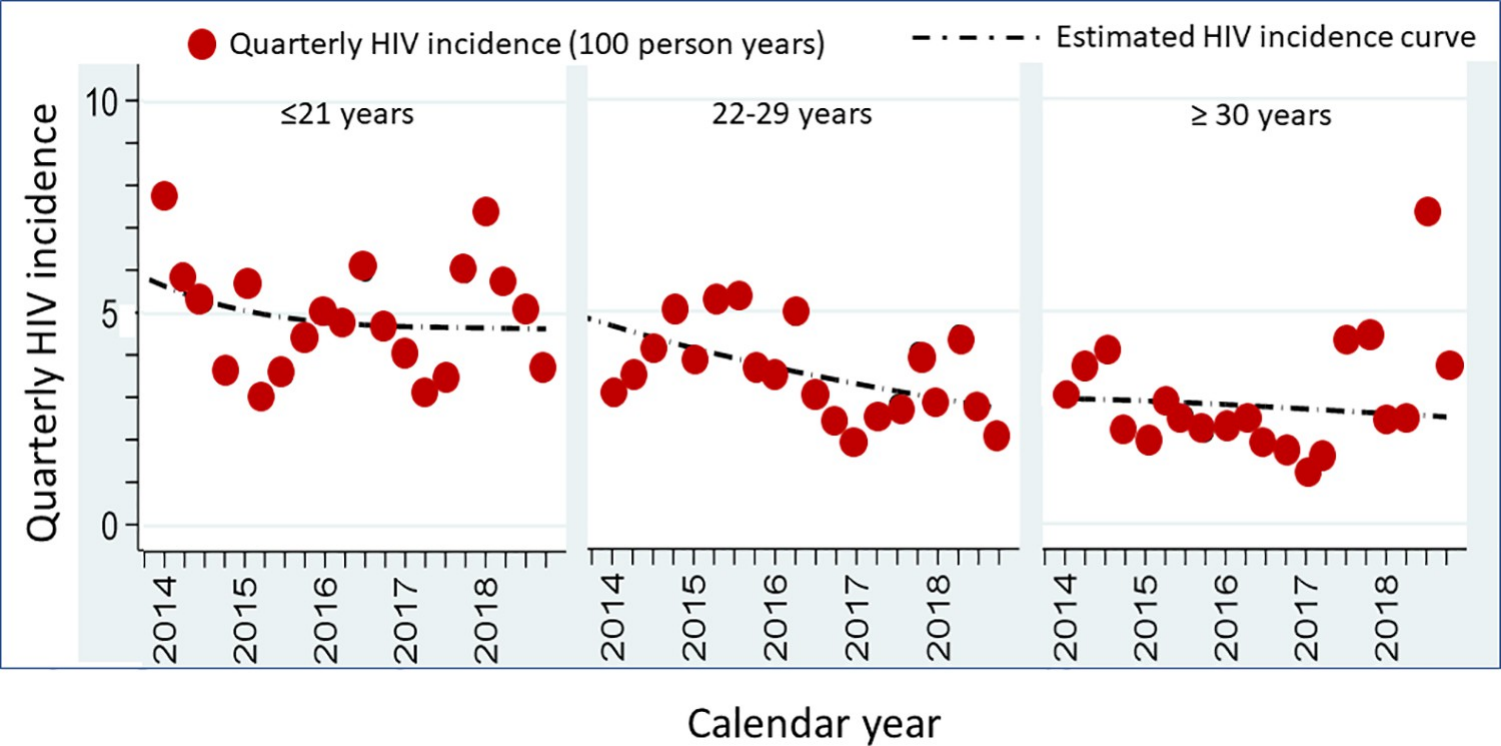
Quarterly HIV incidence among men who have sex with men attending HIV testing and counseling services at the Silom Community Clinic, Bangkok, 2014–2018, by age group.

*HIV incidence estimates from HIV testing sites*. Like HIV prevalence, HIV incidence data were presented for MSM who came in for repeated HIV testing at TRCAC between 2014 and 2018. HIV incidence density in these men was 4.2 per 100 PY in 2014, decreasing to 2.1 per 100 PY in 2018 (Table 3) (N Phanuphak, personal communication). Data were shown from the SCC, where HIV incidence density fell from 4.1 per 100 PY in 2014 to 3.3 per 100 PY in 2018 (Table 3). Age-disaggregated quarterly HIV incidence density data were also available from SCC. HIV incidence was highest in the youngest age group (age ≤21 years) (Fig 3) [39].

**Table 3:** HIV incidence density among men who have sex with men attending HIV testing and counseling services at the Thai Red Cross Anonymous Clinic and Silom Community Clinic, Bangkok 2014–2018.

Venue	Thai Red Cross Anonymous Clinic			Silom Community Clinic		
Calendar year	Number of new HIV infections	Number of person years	HIV incidence density	Number of new HIV infections	Number of person years	HIV incidence density
2014	203	5017	4.2	58	1425	4.1
2015	183	4692	3.9	59	1612	3.7
2016	213	6288	4.5	57	1539	3.6
2017	204	8168	2.5	39	1259	3.1
2018	282	13429	2.1	17	518	3.3

### Transgender women

#### HIV prevalence

**Fig 4:**
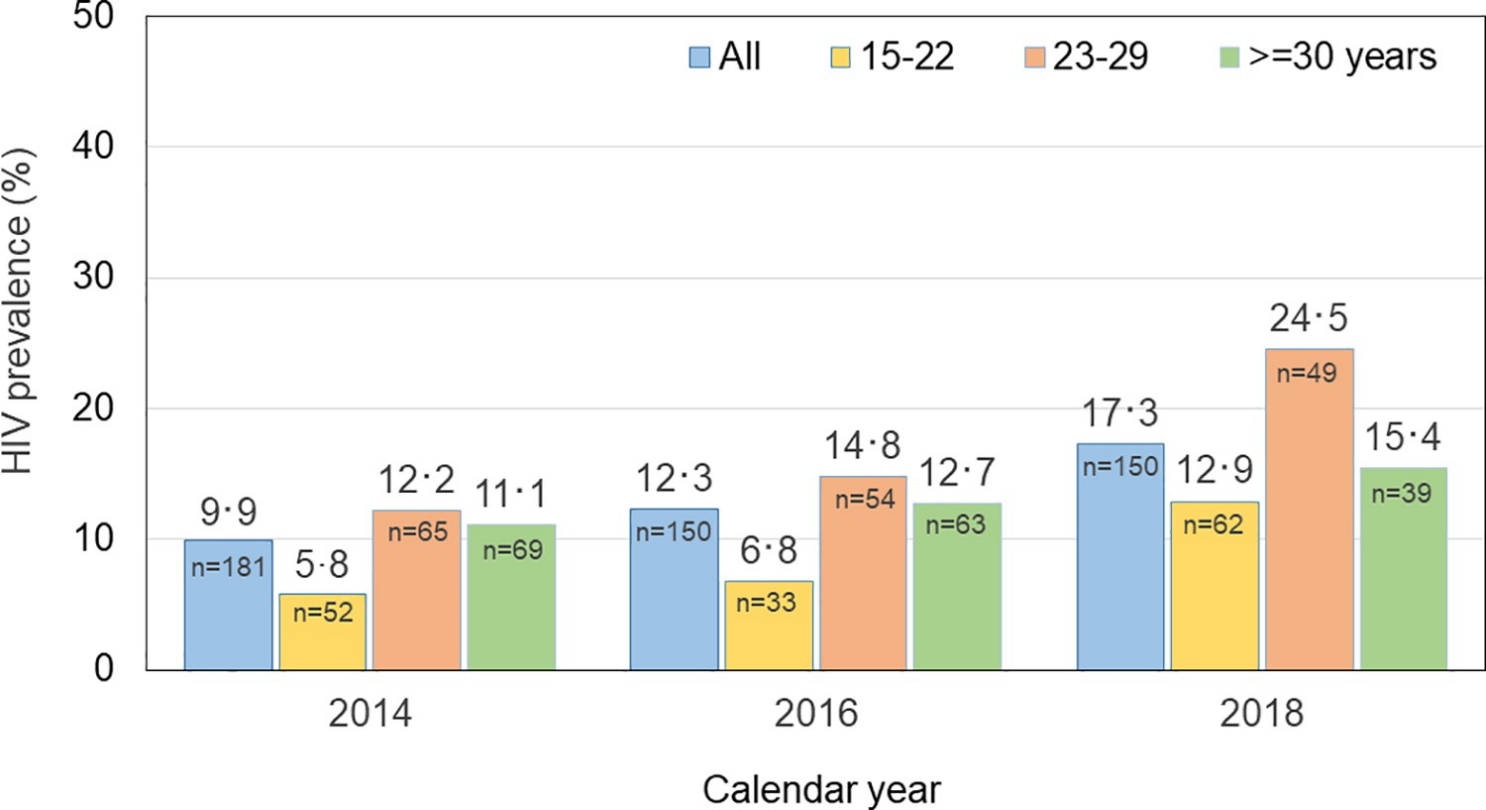
HIV prevalence among transgender women, integrated behavioral and biological surveillance, Bangkok, 2014–2018, by age group.

*HIV prevalence in cross-sectional studies*. Data were presented from IBBS conducted among TGW in Bangkok. In 2014, HIV prevalence was 5.8% among 15- to 22-year-olds, 12.2% among 23- to 29-year-olds and 11.1% among TGW of 30 years and older. Four years later, the prevalence among 15- to 22-year-olds had increased to 12.9%, among 23- to 29-year-olds to 24.5% and among those of 30 years and older to 15.4%. Consequently, overall HIV prevalence among TGW in IBBS increased from 9.9% in 2014 to 17.3% in 2018 [8] (Fig 4).

Among Bangkok TGW enrolled in the Test, Treat and Prevent Program [35], the HIV prevalence was 4.7% (2/43) in those 21 years or younger, 10.5% (6/57) in 22- to-29-year-olds and 4.4% (2/23) in those of 30 years and older. The overall HIV prevalence was 7.3% (9/123) (C Manopaiboon, personal communication).

The Tangerine Clinic is a dedicated health service for transgender people at TRCAC since 2016. The number of TGW clients per year was 466 in 2016, 488 in 2017 and 999 in 2018 [43]. HIV prevalence among these women was 14.6% (66/446) in 2016, 8.0% (36/488) in 2017 and 11.4% (114/999) in 2018 [43].

#### HIV incidence

*HIV incidence estimates from cross-sectional studies*. Using age at start of anal intercourse and HIV infection status at the time of assessment (method described above for young MSM) [40], HIV incidence was estimated among young TGW (≤22 years old) taking part in IBBS from 2014 to 2018 [8, 11]. Estimated HIV incidence density in this population was calculated as 1.5 (3/200) per 100 PY in 2014, 1.4 (2/144) in 2016 and 4.5 (8/178) in 2018 (F van Griensven, personal communication).

*Observed HIV incidence*. HIV incidence data among TGW are limited and were reported at the consensus meeting only by the Tangerine Clinic. The number of TGW who were repeatedly tested for HIV infection at Tangerine increased from 98 in 2016 to 407 in 2018. The HIV incidence density in these women increased from zero (0/36) in 2016 and 2.3 (2/87) in 2017 to 3.3 (6/182) per 100 PY in 2018 [43].

#### HIV consensus voting

After presentation and deliberation of available data the following items were presented for voting consensus (agree/disagree; see S1 Appendix): 1) HIV prevalence and incidence are decreasing among Bangkok MSM; 2) Remaining HIV prevalence and incidence are unacceptably high, especially among young MSM; 3) HIV prevention programming among MSM needs to be intensified, particularly for young MSM; 4) There are no signs of a decrease in HIV prevalence and incidence among TGW; 5) More information is needed to appropriately monitor the HIV epidemic among TGW; 6) At the current rate of new infections in MSM and TGW, Thailand will not be able to reach its goal of ending AIDS by 2030. Consensus was reached among experts and stakeholders regarding all items presented above.

## Conclusions

Decreasing HIV prevalence and incidence were reported among Bangkok MSM from 2014 to 2018. These declines were consistent across all MSM data-sources reviewed during the HIV epidemic consensus process. While there was agreement about these reductions, the meeting ruled that HIV prevalence and incidence remained unacceptably high. Particularly concerning was the HIV incidence among young MSM, which as the cohort ages will limit further declines in HIV prevalence seen in older MSM for years to come. There was a dearth of information about the spread of HIV infection in Bangkok TGW. Until well into the past decade, TGW were commonly merged with MSM and not studied or reported as a separate risk category [44, 45]. In more recent years however, this has started to change, which is also reflected by the data presented in our report. Nevertheless, the number of dedicated studies remains limited, and if disaggregated data regarding TGW were available investigations were usually insufficiently powered to support any conclusions. In this context, meeting participants agreed there were no signs of a decrease in HIV prevalence and incidence in this population.

HIV prevalence and incidence among MSM in Bangkok appear to be decreasing. These decreases may, at least in part, be attributable to the introduction of more effective HIV prevention measures, such as TasP and HIV PrEP [36, 46]. However, HIV prevalence may also decrease when HIV infected individuals are moving out of the population due to premature mortality. Those who have been previously diagnosed may also opt out of repeated IBBS surveys, which can mean that those who consent may underrepresent the true HIV prevalence in the community. While Thailand has made great strides in providing access to ART for the general HIV-infected population [25], the situation in marginalized groups may be different. Stigma and discrimination against Thai MSM and TGW are common and are known to obstruct timely access to HIV treatment and care [47–49].

Another caveat is that HIV prevalence and incidence data were collected using a diverse array of recruitment methodologies in a variety of settings, usually time-location or convenience sampling at venues where MSM and TGW gathered or came for HTC. As a result, it is unknown to what extent findings are representative of Bangkok MSM and TGW communities at large. In addition, HIV laboratory testing procedures may have evolved or been different between study sites, which may negatively affect comparability within and between them. Nevertheless, only information collected from 2014 to 2018 in Bangkok was taken into consideration, allowing the study of trends over time while minimizing geographic, laboratory and methodologic variation. The reported decline in HIV prevalence among Bangkok MSM enrolled in IBBS may also partially result from an increase in the number of young MSM taking part in 2018. Since MSM accumulate HIV infection with increasing age, the HIV prevalence in younger MSM is per definition lower. On the other hand, HIV prevalence already started to decline in 2016, when only few young MSM were included in the survey.

Despite recent progress in reducing the spread of HIV in MSM, there was consensus among experts and stakeholders alike that at the current rate of new HIV infections among MSM and TGW, Thailand will not be able to reach its goal of ending AIDS by 2030 [22]. Although discussion of possible next steps was not within the purview of this HIV epidemic consensus initiative, there are strategies that may contribute to further reductions of HIV infection. For example, increased focus and attention on the uptake of HIV testing, ART and PrEP initiation, retention and adherence and reduction of stigma and discrimination in the health care setting are necessary. Data collected during IBBS in recent years indicate that only about half of MSM and TGW in Bangkok had ever been HIV tested [8]. This, in combination with a significant need to further scale the uptake, retention and adherence of ART and PrEP [46, 50], will contribute to ongoing HIV transmission. Increased access and better retention and adherence support methods are therefore needed, especially for younger MSM and TGW. Long-acting injectable PrEP has recently been found superior to oral daily PrEP in MSM and TGW [51]. While injectable PrEP may help to offset decreased protection resulting from poor adherence, acceptability and feasibility of this approach is still under investigation [52, 53]. Alternative HIV testing approaches, such as home-based and online-supported testing [37, 54], may help to reach populations known to have low uptake and higher HIV incidence, such as younger MSM and TGW, and those in sexual networks of HIV infected MSM and TGW. Widening and improvement of partner tracing and testing services may particularly be beneficial in networks of MSM and TGW with acute or recent HIV infection. HIV viral load and risk of onward transmission are known to be highest during the early stages of infection, which may be reduced by early diagnosis and immediate initiation of ART.

Risk factor studies have shown a crucial role of methamphetamine use for sexual pleasure in HIV transmission among Thai MSM and TGW [55, 56]. Currently, methamphetamine use is criminalized in Thailand, which makes access to drug using MSM and TGW more difficult. Decriminalization and treatment of methamphetamine use may help to widen options for prevention and treatment over penalizing measures. Finally, greater HIV prevention efforts should also address social and structural determinants of risk not modifiable at the individual level, such as equal rights, legal recognition, and prevention of discrimination and stigma of MSM and TGW in health-care settings.

In conclusion, the Bangkok HIV epidemic consensus initiative was able to mobilize a variety of different data sources allowing exceptional insights into the ongoing HIV epidemic in the Thai capital. While recent biomedical HIV prevention approaches may have had some effect in containing the spread of HIV infection among MSM and TGW, their overall impact was found insufficient to control the epidemic. Finally, this initiative proved valuable in building consensus on complex epidemiologic and program data among experts and stakeholders and should be considered for use in other metropolitan areas heavily burdened by HIV.

## Supporting information

S1 Appendix Bangkok HIV consensus development initiative voting items. (DOCX)
