# Supplementary material for: HIV prevalence and incidence among men who have sex with men and transgender women in Bangkok, 2014–2018: Outcomes of a consensus development initiative
Source: PLoS One. 2022 Jan 21;17(1):e0262694. doi: 10.1371/journal.pone.0262694 (PMC8782340; doi:10.1371/journal.pone.0262694)
Supplement: S1 Appendix — (DOCX) [file pone.0262694.s001.docx]

| **Bangkok HIV consensus development initiative voting items** | | |
| --- | --- | --- |
| Item number | Statement (item) | Answer (click whether you agree or disagree) |
| 1 | HIV prevalence and incidence are decreasing among Bangkok MSM | Agree/disagree |
| 2 | Remaining HIV prevalence and incidence are unacceptably high, especially among young MSM | Agree/disagree |
| 3 | HIV prevention programming among MSM needs to be intensified, particularly for young MSM | Agree/disagree |
| 4 | There are no signs of a decrease in HIV prevalence and incidence among TGW | Agree/disagree |
| 5 | More information is needed to appropriately monitor the HIV epidemic among TGW | Agree/disagree |
| 6 | At the current rate of new infections in MSM and TGW, Thailand will not be able to reach its goal of ending AIDS by 2030 | Agree/disagree |
| MSM, men who have sex with men; TGW, transgender women | | |
